# Isolation and Characterization of Lactic Acid Bacteria from Fermented Milk Produced in Jimma Town, Southwest Ethiopia, and Evaluation of their Antimicrobial Activity against Selected Pathogenic Bacteria

**DOI:** 10.1155/2022/2076021

**Published:** 2022-12-13

**Authors:** Tigistu Goa, Getenet Beyene, Mekidim Mekonnen, Kasahun Gorems

**Affiliations:** ^1^College of Natural and Computational Science, Department of Biology, Wolkite University, Ethiopia; ^2^Faculty of Health Sciences, School of Medical Laboratory Sciences, Department of Medical Microbiology, Jimma University, Ethiopia; ^3^St Paul's Hospital Millennium Medical College, Addis Ababa, Ethiopia

## Abstract

**Background:**

Raw milk is usually contaminated with pathogenic bacteria. Fermentation of milk is important to inhibit the growth of contaminants, spoilage, and pathogenic bacteria. The objective of this study was to isolate lactic acid bacteria from fermented milk and evaluate their antimicrobial activity against selected pathogenic bacteria.

**Methods:**

Laboratory-based experimental study design was conducted from May-July, 2021.Three samples of Ergo (each of 250 ml) were collected from Jimma town. Lactic acid bacteria (LAB) isolates were identified through integrated phenotypic techniques. Further identification was conducted through using API 50 CHL strips. Antimicrobial activities (AMAs) of LAB isolates were tested against clinical isolates of *E. coli*, *S. aureus*, and *Salmonella* spp. using agar well diffusion method. The data were analyzed by using SPSS software version 21 and Microsoft Excel spreadsheet. Tables and figures were applied to describe characteristics of data.

**Results:**

Twelve LAB isolates were identified. Those LAB isolates include six *Lactococcus lactis* subsp. *lactis*, *Lactobacillus acidophilus* (2), *Lactiplantibacillus plantarum* (1), *Limosilactobacillus fermentum* (2), and *Leuconostoc lactis* (1). Based on primary screening of LAB, isolates/strains ESCIa, ESBIa, and ESCIc show strong AMA against *S. aureus*, *E. coli*, and *Salmonella* spp. The CFS of ESCIc showed the highest AMA against *S. aureus* and *Salmonella* spp. with a zone of inhibition of 14.12 ± 1.6 mm and 12.9 ± 3.6 mm, respectively, while ESBIa showed the highest AMA against *E. coli* with a zone of inhibition of 13.5 ± 2.1 mm. The CFSs of selected LAB strains were heat tolerant at varying temperatures up to 100°C. The CFSs of selected LAB strains were inactivated by proteinase enzymes, but they are not inactivated with amylase enzymes. *Conclusions and Recommendation*. All 12 LAB isolates exhibited antimicrobial activity against tested bacterial strains. *Lactobacillus* isolates showed the highest antagonistic activity on tested indicator strains. Thus, they are possible alternatives to antibiotics in the era of antimicrobial resistance. *S. aureus* was the most sensitive to antimicrobial effects/agents of selected LAB isolates. Consumption of fermented foods is advisable since they support the growth of healthy GIT microbiota. Fermentation serves as biopreservation of food. However, analysis of probiotic features and *in vivo* probiotic effects of those LAB isolates will be subject of future research/study.

## 1. Background

Lactic acid bacteria (LAB) are a wide group of gram-positive, nonspore-forming, catalase negative, and aerotolerant bacteria that includes a large number of genera (rods and cocci), [1]. LAB are microorganisms which cause fermentation of foodstuffs. They are classified based on their morphology, mode of glucose fermentation, growth at various temperatures, and tolerance to salt. In addition, fatty acid composition and motility are used for identification of LAB [2].

After searching for the terms “LAB” and “Food”, “LAB” and “Gut”, and “LAB” and “Environment” in scientific database Scopus: 11,800, 1,500, and 1,700 documents have been retrieved, respectively. Those numbers indicate that food is the most widely studied environment and favored niche for lactic acid bacteria proliferation [3]. Food and Agricultural Organization (FAO), World Health Organization (WHO), and International Scientific Association for Probiotics and Prebiotics (ISAPP) agreed upon the definition of probiotics as “they are defined as harmless live normal flora which can provide/confer health benefit(s) to the host, when administered in adequate amount” [4, 5].

Probiotics have attracted extraordinary attention in the scientific community with an escalating number of investigations/studies. Thanks to microbiome research which changed the perception about microorganisms beyond disease-causing agents to appreciate their role in disease management [6]. Some of the importance of probiotics has been documented including reestablishing the composition and function of the microbial community, preventing the growth of unwanted pathogens, and enhancing/improving the gastrointestinal tract function by providing vitamins and amino acids to the host. Therefore, probiotic microorganisms play a significant role in the food, feed, dairy, and fermentation industries for the purpose of nonpharmacological intervention for maintaining the health of the host/consumers [7].

Now, in the 21^st^ century, antibiotic resistant bacterial infections are on the rise, while the rate of discovery of new antibiotics decreases [8]. In addition, the biggest fear in the future will be the limited effectiveness of antibiotics against bacterial pathogens [9]. Globally, more than 1,000,000 people die because of antibiotic resistant pathogens. If solutions are not found, number of death due to antibiotic resistant pathogens might be elevated to ten million by 2050. Developing new drugs and its characterization is a complex process, expensive, and requires a long period of time [10]. Currently, searching for new antimicrobials to combat the risks of antimicrobial resistance is the prioritized concern of the WHO [11]. Therefore, researchers and scholars around the world are devoted to finding and exploring an effective and alternative biological antimicrobial agent to control and eradicate the burden of drug-resistant pathogenic bacteria [12]. Antibiotic treatment not only affects pathogenic bacteria but also nontargeted commensal intestinal microbial communities were disturbed and led to intestinal microbiota dysbiosis; mainly antibiotic-associated diarrhea is one of the intestinal problems [13]. So, using probiotics to restore the intestinal microbiota will be an alternative to antibiotics.

Pathogenic and food spoilage bacteria must be controlled to ensure food quality. The side effects of chemical preservatives that prevent growth of contaminants and extend the shelf life of food pose a challenge to modern food-processing technology. Thus, interest in “Green technology” representing a new ways of food processing and the use of microbial-derived metabolites as biopreservatives became an emerging technology [14]. Food preservation techniques are necessary to extend the shelf life of foodstuffs via killing or inactivating the contaminants and pathogens. However, mostly due to the specific national culture, in some countries, raw food is utilized without applying any preservation techniques. For example, people in Ethiopia have a trend of consuming raw milk [15]. Thus, reduction of milk contamination is not possible yet [16].

In Jimma town, milk and its products are dominantly used for consumption and as source of income for producers [17]. Therefore, controlling raw milk contamination will be an important consideration in the food business. The use of LAB strains or their antimicrobial products to inhibit pathogenic bacteria in the milk environment was introduced to the concept of preservation. LAB produce different kinds of antimicrobial compounds like bacteriocins, hydrogen peroxide, and organic acids such as lactic acid. Among them, bacteriocins are mainly used in the food industry to prevent food spoilage and foodborne diseases. Bacteriocins have many characteristics such as nontoxicity, inactivation by digestive tract-related proteases, genetically engineered, served as natural food preservatives, and they are generally recognized as safe for use [9, 18, 19].

The use of antimicrobial metabolites/peptides to extend the shelf life of milk and milk products through antagonizing and killing food spoilage and pathogenic bacteria has given an interesting result worldwide [14]. Therefore, the main objective of this study was to isolate LAB from naturally fermented cow's milk (Ergo) and to evaluate their antimicrobial activity against selected bacterial pathogens that cause foodborne diseases.

## 2. Methods

### 2.1. Study Design, Period, and Area

The study was conducted in Jimma town, located 355 km southwest of Addis Ababa, Ethiopia. From a climatic point of view, ample/abundant rainfall makes this area suitable for agricultural production. Livestock production is an important activity in this area, and milk is produced in small dairy farms established in the city and then sold to collection centers, vendors, and consumers [20]. Laboratory-based experimental study design was conducted from May-July, 2021; samples of fermented milk products were collected for isolation of lactic acid bacteria and evaluation of their antimicrobial activity against selected food pathogenic bacteria: *Escherichia coli, Salmonella* species, and *Staphylococcus aureus*.

### 2.2. Study Participants and Data Collection

In Jimma town, a total of eight milk collection centers were visited. Five collection centers were excluded from the study because they are raw milk sellers. Three of them were included in the study to collect Ergo samples. Since a few samples are enough according to laboratory-based experimental study design, for this study, three samples of Ergo (each of 250 mL) were collected from three collection centers in Jimma town.

### 2.3. Milk Sample Collection and Transportation

Before conducting the study, an agreement of written consent was signed with milk sellers, after being informed about the reason for conducting the research. To avoid contamination during sample collection, a maximum sterile working condition was implemented. The samples were collected in a sterile cup bottle and labeled with the date of collection. All Ergo samples (3) were transported to the microbiology laboratory of the School of Medical Laboratory Sciences of Jimma University immediately after sampling on ice, where the laboratory analysis was carried out.

### 2.4. Laboratory Analysis of Fermented Milk Samples

#### 2.4.1. Plating and Isolation of Lactic Acid Bacteria from Fermented Milk

To isolate lactic acid bacteria from Ergo, serial dilutions were made by adding first 1 mL of fermented milk to 9 mL of sterile distilled water (from 10^−1^ up to 10^−7^) [21]. Later on, 0.1 mL of the last serial dilutions was taken and spread on to the surface of predried de Man Rogosa Sharpe (MRS) agar (Oxoid, Basingstoke, Hampshire, England) media. After inoculation, the plates were incubated under anaerobic conditions using an anaerobic candle jar at 37°C for 48 hours [1]. After successful growth of LAB on MRS agar, morphologically varying/distinct colonies were further subcultivated/purified by streaking on new MRS agar plates using sterile-inoculating needles and incubated at 37°C for 24-48 hours inside the incubator [21].

#### 2.4.2. Identification of Lactic Acid Bacteria Isolated from Fermented Milk

Lactic acid bacteria isolated from Ergo were identified based on morphological, physiological, and biochemical tests [1]. Based on the result of identification by preliminary tests, isolates were categorized into various genera. Furthermore, carbohydrate fermentation test was conducted by using analytical profile index (API 50 CHL) strips [22]. LAB isolates were identified based on the pattern of sugar utilization and also supported Bergey's Manual of Determinative Bacteriology.

### 2.5. Morphological Examination

#### 2.5.1. Gram Staining

Gram staining was done using standard procedure and gram-positive bacilli/cocci were chosen for further characterization [23].

### 2.6. Physiological Examination

#### 2.6.1. Growth at Different Environmental Temperature

The growth of bacterial isolates at different temperatures (15 and 45°C) was the main criteria for identifying LAB, which are *bacilli* isolates, while growth at 10 and 45°C was used for identifying cocci LAB isolates [24]. For this purpose, 10 mL of nutrient broth which is supplemented with 1% glucose was used for culturing lactic acid bacteria isolates. The growth of the isolates was analyzed after 24 hours by observing the turbidity [25].

#### 2.6.2. Growth at Different NaCl Concentration

The growth of bacteria at 4 and 6.5% salt concentrations was the main criterion for identifying *bacillus* isolates, while cocci isolates were able to grow at 2 and 4% NaCl concentrations [24]. Among the cocci isolates, the growth of *Enterococcus* is also initiated to grow at 6.5% NaCl concentrations [26]. Purified colony of LAB was inoculated first into 10 mL of nutrient broth and grown overnight. Well-grown culture was used for inoculation (1% inoculum) of 10 mL of nutrient broth supplemented with 2%, 4%, and 6.5% NaCl concentrations. The growth of isolates after 24 hours of incubation was determined by visual observing the turbidity [25].

### 2.7. Biochemical Identification

#### 2.7.1. Catalase Test

Catalase enzymes break down hydrogen peroxide into oxygen (which is seen/visualized as the formation of bubbles) and water molecules (2*H*_2_*O*_2_⟶2*H*_2_*O* + *O*_2_). The catalase test was conducted by adding a drop of 3% solution of hydrogen peroxide to a glass slide on which a colony of bacteria was applied of a 24 hour-old culture of each isolate (or directly on the Petri dish). NB: catalase negative bacteria were subjected to further examination [27].

#### 2.7.2. Gas Production from Glucose

The production of carbon dioxide gas from glucose was important to determine the homo-heterofermentative nature of an isolate. Pure culture isolates of lactic acid bacteria were inoculated into a 10 mL nutrient broth containing an inverted Durham tube and incubated at 37°C for 48 hours [23]. In the case of homofermentative LAB, there is no production of gas and in the case of heterofermentative LAB, there is production of gas inside Durham tube.

#### 2.7.3. Triple Sugar Iron Agar

Triple Sugar Iron (TSI) agar was inoculated with pure culture of the isolates by stabbing through the center of the medium to the bottom of the tube and then streaked to the surface of the slant. Lastly, the tube was incubated at 37°C for 24 hours. LAB isolates fermented three sugar units such as glucose, sucrose, and lactose within the TSI medium and produces acids which are indicated by color changes to yellow [28]. In this study, the LAB isolates were positive as they fermented three sugars by showing a yellow color, while the isolates were negative for hydrogen sulfide (H_2_S) production.

#### 2.7.4. Simmons Citrate Agar

Simmons citrate agar was used to distinguish whether lactic acid bacteria are able to use citrate as its sole carbon source. Simmons citrate agar was prepared and dispensed into clean test tubes, sterilized, and allowed to solidify in inclined position. Using a sterile-inoculating loop, a small amount of well-purified colony of lactic acid bacteria was taken from a 24 hours old culture and streaked into the slant of Simmons citrate agar and was incubated for 24 hours at 37°C. Those LAB isolates that changed color from green (original color of prepared medium) to blue or yellow were considered positive [29].

#### 2.7.5. Test (Indicator) Bacteria

To detect the antimicrobial activity of LAB isolates, pathogenic bacteria such as *S. aureus, E. coli*, and *Salmonella* species were used [30]. The used pathogenic indicator bacteria were obtained from the microbiology laboratory of Jimma University Medical Center and represent clinical isolates (four isolates/strains per species) that have been biochemically characterized along with antimicrobial susceptibility patterns.

### 2.8. Antimicrobial Activity of Lactic Acid Bacteria against Indicator Bacteria

#### 2.8.1. Primary Screening

Previously conducted research focused on identifying and characterizing the individual antimicrobial mediators in simple in vitro systems. But little information is known about the combined effect of antimicrobial compounds [31]. The primary screening was conducted to establish combined effect of antimicrobial compounds. Antimicrobial activity of lactic acid bacteria was conducted by using agar well diffusion antimicrobial assay [32]. The pathogenic indicator bacterial strains were inoculated into a test tube containing 5 mL of nutrient broth and incubated at 37°C for 24 h with aeration (shaking at 180 rpm) to obtain active cultures. The entire surfaces of each nutrient agar plate were swabbed with indicator bacteria by using sterile cotton swabs or spread with sterilized glass spreader [33]. Four wells of 6 mm diameter were made using sterile yellow Pasteur pipette tips. Then, 100 microliters of LAB isolates of overnight culture in nutrient broth was added into the wells. The inoculated media were placed inside an anaerobic candle jar and incubated for 24 hours at 37°C. After incubation, the plates were observed for a zone of inhibition (ZOI) around the well. The diameter of the inhibition zone was measured in mm by using calipers and a clear zone of 1 mm or more was considered as positive inhibition [1]. The diameter (in mm) of the zone of growth inhibition around each well was evaluated considering the total zone minus the diameter of the Pasteur pipette tips [34]. The LAB isolates that showed maximum zone of inhibition against indicator bacteria were further characterized. Those isolates were subcultured on MRS agar and stored in 15% Tryptic Soy Broth glycerol solution for secondary screening such as antibacterial compound extraction and its characterization [14].

### 2.9. Secondary Screening

#### 2.9.1. Preparation of Cell-Free Supernatant

The cell-free supernatants (CFSs) were prepared based on methods by Assefa [27], with some modification. LAB isolates were transferred into 5 mL of nutrient broth and incubated at 37°C for 24 hrs. The broth culture was centrifuged at 10,000 rpm at 4°C for 20 minutes to get the supernatant; then, it was neutralized to pH 7 by 1 M NaOH to exclude the inhibitory effect of organic acid. Antimicrobial activity of CFS was conducted according to the method used by Al-Allaf et al. [35].

#### 2.9.2. Antibacterial Activity of CFS after Treating them with Heat

For testing temperature stability of antimicrobial molecules produced by selected LAB strains, 5 mL of cell-free supernatant was heated at 30°C, 60°C, and 80°C inside a water bath for 15 minutes. Antimicrobial activity of heat-treated CFS against indicator bacteria was performed by agar well diffusion method; positive control was nontreated CFS [34].

#### 2.9.3. Antibacterial Activity of CFS after Treatment with Enzyme

For testing sensitivity of antimicrobial molecules produced by selected LAB strains on different enzymes, equal volumes of neutralized CFS and the enzymes (3 mg/mL in two times reaction buffer), such as amylase, trypsin, and pepsin were incubated at 37°C for 2 hrs. [36]. Antimicrobial activity of enzyme-treated CFS against indicator bacteria was performed by agar well diffusion method; positive control was nontreated CFS in enzyme reaction buffer.

### 2.10. Statistical Analysis

Statistical analyses were conducted using Statistical Package for the Social Sciences software version 21 and Microsoft Excel spreadsheet. Results were presented as mean values ± standard deviation. Then the data were analyzed and compared statistically using one-way ANOVA and followed by Tukey post hoc test. Data which had *P* < 0.05 were statistically significant. Descriptive statistics such as tables and figures were applied to describe characteristics of data.

## 3. Results

### 3.1. Isolation of Lactic Acid Bacteria from Ergo Samples

Lactic acid bacteria (LAB) were isolated from three (A, B, and C) traditionally fermented milk samples, locally called “Ergo” samples (ES). The isolation was accomplished by following a routine microbiological process and inoculation on solid medium. After inoculation of appropriate dilutions of Ergo samples (ESA, ESB, and ESC) on de Man Rogosa Sharpe agar (MRS agar) media, successfully cultured LAB isolates (12) were subjected for integrated phenotypic identification (morphological, physiological, and biochemical tests).

### 3.2. Identification of Lactic Acid Bacteria from Ergo Samples

**Figure 1:**
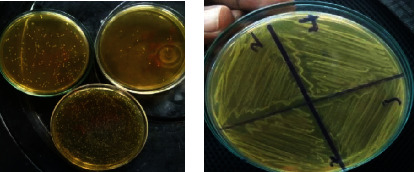
(a) Lactic acid bacteria on MRS agar; (b) LAB after purification on MRS agar.

After the growth of LAB, macroscopic observation of the colonies on the surface of MRS agar Petri plate was performed (Figure 1(a)) to select a number of different colonies. All preselected colonies were creamy after repurification (Figure 1(b)).

Shape, size, and coloration of the LAB isolates were evaluated after the gram-stained bacterial smear; all of LAB isolates were gram positive, five of the 12 lactic acid bacteria isolates were rods (ESBIa, ESCIa, ESCIb, ESCIc, and ESCId) while seven were cocci (ESAIa, ESAIb, ESAIc, ESAId, ESBIb, ESBIc, and ESBId). Even though LAB isolates such as ESAIa, ESAIb, ESAIc, ESAId, ESBIc, and ESBId were determined to belong to *Lactococcus* genera, they are different from each other by their cellular appearance observed after microscopic analysis. Among cocci, ESBIb also showed different cellular appearance. LAB isolates such as ESBIa, ESCIa, ESCIb, ESCIc, and ESCId that belong to *Lactobacillus* genera also have different cellular appearances (Table 1).

The gram-positive cocci and rod-shaped bacteria, which were subcultured on MRS agar were further characterized using catalase test, Simmons citrate utilization test, carbohydrate fermentation, hydrogen sulfide and gas production, ability of growth at different temperature, and salt concentrations (Table 2). According to the results obtained (shown in Table 2), all (12) isolates were catalase negative, six isolates (ESBIa, ESBIb, ESCIa, ESCIb, ESCIc, and ESCId) were positive for gas production (heterofermentative lactobacilli and leuconostoc), while the other six isolates were negative for gas production (homofermentative lactococci).

The Simmons citrate utilization test indicated that two isolates (ESCIa and ESCIb) were positive, while other ten isolates (ESAIa, ESAIb, ESAIc, ESAId, ESBIa, ESBIb, ESBIc, ESBId, ESCIc, and ESCId) were negative. The carbohydrate fermentation and hydrogen sulfide production test showed that all isolates are glucose, lactose, and sucrose fermenters and were negative for hydrogen sulfide production. Isolates ESAIa, ESAIb, ESAIc, ESAId, ESBIb, ESBIc, and ESBId were capable to grow at 10°C, while isolates ESBIa, ESCIa, ESCIb, ESCIc, and ESCId were unable to grow at 10°C. All LAB isolates were able to grow at 15°C, but isolates ESBIa, ESCIa, ESCIb, and ESCId were able to grow at 45°C, while ESAIa, ESAIb, ESAIc, ESAId, ESBIb, ESBIc, ESBId, and ESCIc isolates did not grow at 45°C. Ability to grow at different salt concentrations indicated that all LAB isolates were capable to grow at 2% and 4% NaCl concentration. Growth at 6.5% NaCl concentration was observed by isolates ESBIa, ESBIb, ESCIa, ESCIb, ESCIc, and ESCId, while isolates ESAIa, ESAIb, ESAIc, ESAId, ESBIc, and ESBId (Table 2) were not able to grow at 45°C.

Based on morphological characteristics (Table 1), physiological, and biochemical characteristics examined (Table 2), the isolated LAB were presumptively identified as *Lactococcus* genera (six isolates: ESAIa, ESAIb, ESAIc, ESAId, ESBIc, and ESBId), *Lactobacillus* genera (five isolates: ESBIa, ESCIa, ESCIb, ESCIc, and ESCId), and *Leuconostoc* genera (one isolate: ESBIb).

Based on the result of identification by API 50 CHL test strips (Table 3), the LAB isolates such as ESAIa, ESAIb, ESAIc, ESAId, ESBIc, and ESBId belong to *Lactococcus lactis* subsp. *lactis*. One isolate (ESBIb) was *Leuconostoc lactis*. While the other isolates such as ESBIa and ESCIa were *Lactobacillus acidophilus*. But ESCIb belonged to *Lactiplantibacillus plantarum*. The isolates such as ESCIc and ESCId were *Limosilactobacillus fermentum*.

### 3.3. Antimicrobial Activity of LAB Isolates against Indicator Bacteria

**Figure 2:**
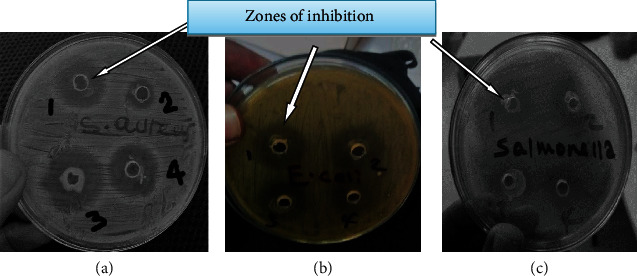
Combined effects of antimicrobial compounds of LAB isolates against (a) *S. aureus*, (b) *E. coli*, and (c) *Salmonella* spp. Arrows indicate zones of inhibition. The numbers such as 1, 2, 3, and 4 show the representative LAB isolates with different sizes of zones of inhibition.

Antimicrobial activity of 12 lactic acid bacteria isolates was performed by using agar well diffusion antimicrobial assay on the basis of their capacity to suppress/inhibit the growth of indicator bacteria such as *Staphylococcus aureus*, *Escherichia coli*, and *Salmonella* species. The LAB isolates displayed antagonistic activities against indicator bacteria used in this study. Antibacterial activity of the LAB isolates was determined based on the appearance of a clear zone of inhibition around the wells marked from one to four (Figures 2(a)*–*2(c)). In addition, on Figure 2, it can be noticed the growth of LAB in the wells and even around the wells.

**Figure 3:**
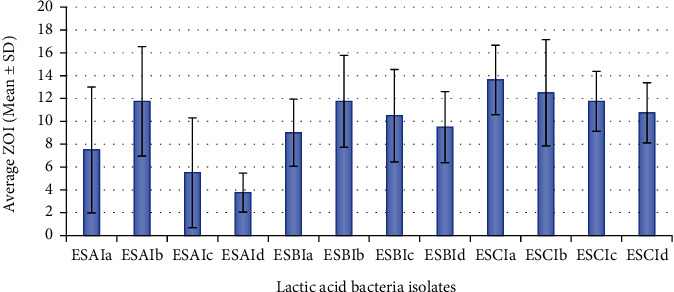
Average inhibition zones of LAB isolates against *S. aureus.*

The most active against *Staphylococcus aureus* was isolate ESCIa with an average inhibition zone of 13.6 ± 3.1 mm, followed by ESCIb with an average inhibition zone of 12.5 ± 4.6 mm, and ESCIc, ESAIb, ESBIb, ESCId, ESBIc, ESBId, ESBIa, ESAIa, ESAIc, and ESAId with average inhibition zones of 11.7 ± 2.6, 11.7 ± 4.8, 11.7 ± 4.1, 10.7 ± 2, 10.5 ± 4.1, 9.5 ± 3.1, 9 ± 2.9, 7.5 ± 5.5, 5.5 ± 4.8, and 3.7 ± 1.7 mm, respectively (Figure 3).

**Figure 4:**
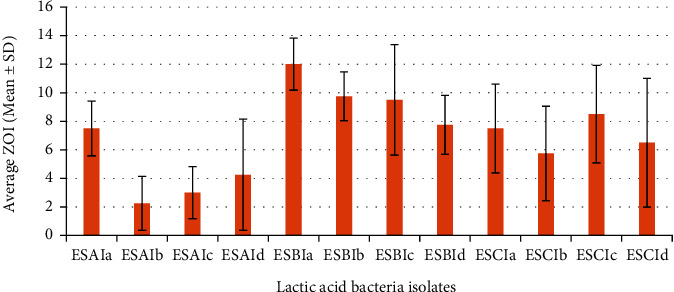
Average inhibition zones of LAB isolates against *E. coli.*

For *Escherichia coli*, the most active lactic acid bacteria isolate was ESBIa with an average inhibition zone of 12 ± 1.8 mm, followed by ESBIb with an average inhibition zone of 9.7 ± 1.71 mm, and ESBIc, ESCIc, ESBId, ESCIa, ESAIa, ESCId, ESCIb, ESAIc, ESAId, and ESAIb with average inhibition zones of 9.5 ± 3.9, 8.5 ± 3.4, 7.7 ± 2, 7.5 ± 3.11, 7.5 ± 1.9, 6.5 ± 4.5, 5.7 ± 3.3, 3 ± 1.8, 4.25 ± 3.9, and 2.2 ± 1.9 mm, respectively (Figure 4).

**Figure 5:**
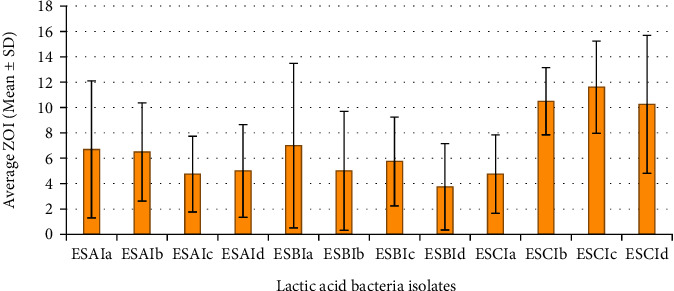
Average inhibition zones of LAB isolates against *Salmonella* spp.

The most active lactic acid bacteria isolate against *Salmonella* species was ESCIc with an average inhibition zone of 11.6 ± 3.6 mm, followed by ESCIb with an average inhibition zone of 10.5 ± 2.6 mm, and ESCId, ESBIa, ESAIa, ESAIb, ESBIc, ESAId, ESBIb, ESAIc, ESCIa, and ESBId with average inhibition zones of 10.2 ± 5.4, 7 ± 6.5, 6.7 ± 5.4, 6.5 ± 3.9, 5.7 ± 3.5, 5 ± 3.6, 5 ± 4.7, 4.7 ± 3, 4.7 ± 3.1, and 3.7 ± 3.4 mm, respectively (Figure 5).

Average inhibition zones produced by each of LAB species were compared and statistically significant difference was observed between the means of groups compared by using one-way ANOVA (Table 4). ANOVA table does not show which mean is significantly different from the other. So, in order to identify exactly which significantly differing, a Tukey post hoc test was conducted (Table 5).

### 3.4. Antimicrobial Activity of CFS of LAB against Indicator Bacteria

**Figure 6:**
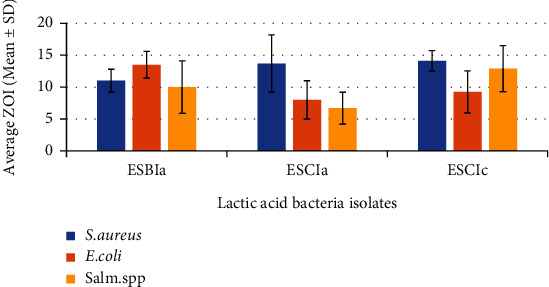
Average inhibition zones of antimicrobial activity of CFS of LAB isolates against indicator bacteria.

Average ZOI of CFS of ESBIa (*Lactobacillus acidophilus*) against *S. aureus*, *E. coli*, and *Salmonella* spp. was 11 ± 1.8, 13.5 ± 2.1, and 10 ± 4.1 mm, respectively. Similarly, average ZOI of ESCIa (*Lactobacillus acidophilus*) against *S. aureus*, *E. coli*, and *Salmonella* spp. was 13.7 ± 4.5, 8 ± 3, and 6.7 ± 2.5 mm, respectively. Average ZOI of ESCIc (*Limosilactobacillus fermentum*) against *S. aureus*, *E. coli*, and *Salmonella* spp. was 14.12 ± 1.6, 9.25 ± 3.3, 12.9 ± 3.6 mm, respectively (Figure 6).

**Figure 7:**
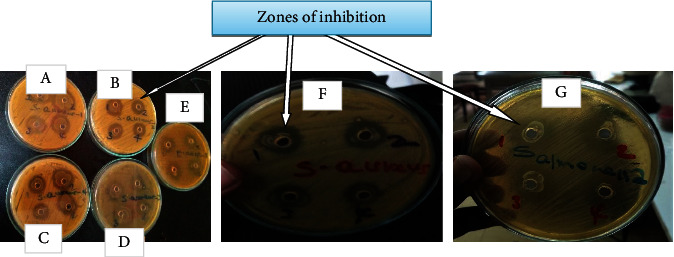
Evaluation of AMA of CFS of LAB isolates against *S. aureus* (A, B, C, D, and F), *E. coli* (E), and *Salmonella* spp. (G). Arrows indicate zones of inhibition. The numbers such as 1, 2, 3, and 4 show the representative LAB isolates with different sizes of zones of inhibition.

Antimicrobial activity of CFS of selected LAB isolates (ESBIa, ESCIa, and ESCIc) observed by showing zone of inhibition around the well (Figure 7). As we observed in the figure, the growth of LAB isolates is also observed inside the well and around the well.

### 3.5. Antibacterial Activity of CFS after Treating them with Heat

According to (Table 6), CFS of three selected LAB isolates was heat-treated at 30°C, 60°C, 80°C, and 100°C for 15 minutes. Antibacterial activity was stable when the CFS heated at temperatures ranging from 30°C to 100°C.

According to (Table 7), antibacterial activity of CFS of three selected LAB isolates was not affected by amylase enzymes. However, the inhibitory activity of ESBIa and ESCIc was affected by pepsin, while the inhibitory activity of ESCIa was affected by trypsin.

All of the *E. coli* isolates were sensitive to tetracycline (75%), ampicillin (50%), cefazolin (25%), ceftriaxone (25%), and ciprofloxacin (25%). While *Salmonella* species isolates showed 100% susceptibility to ceftriaxone, imipenem, and tetracycline, respectively. Similarly, clinical isolates belong to *S. aureus* were susceptible to clindamycin (100%), gentamicin (100%), doxycycline (75%), tetracycline (50%), erythromycin (50%), and penicillin (25%). Among the clinical isolates, *E. coli* isolates showed 100% resistance to gentamicin; *Salmonella* species showed 100% resistant to imipenem, and *S. aureus* isolates showed 75% resistant to penicillin (Table 8).

## 4. Discussion

The central issue of fermenting milk is to extend its shelf life and to preserve the nutritious component of the milk. The presence of fermentative lactic acid bacteria is crucial to the intrinsic properties of fermented food products [37]. Isolation and identification of lactic acid bacteria from their naturally occurring habitat are an important consideration for scientific and commercial purposes. We considered that LAB natural isolates are an exceptional source of new antimicrobial molecules [38]; we directed our study to isolate as many different strains as possible from special “Ergo-” fermented milk products and tested their ability to synthesize antibacterial molecules.

All twelve lactic acid bacteria isolated from “Ergo-” fermented milk products were identified and investigated for their antimicrobial activity against indicator bacteria through agar well diffusion method. All twelve isolates of LAB exhibited antimicrobial activity with varying diameter of the inhibition zone on selected pathogenic bacteria isolated from clinical samples: *Staphylococcus aureus, Escherichia coli*, and *Salmonella* spp.

In this study, *Lactobacillus*, *Lactococcus*, and *Leuconostoc* genera were identified from local-fermented milk products (Ergo), which is similar with results obtained in the study of Burkina Faso [39] on fermented milk with the isolation of LAB genera such as *Lactobacillus*, *Lactococcus*, and *Leuconostoc* isolates. Similarly, Zahara and Zinewi [40] indicated that Ergo fermentation is carried out by lactic acid bacteria belonging to different genera such as *Lactobacillus*, *Lactococcus,* and *Leuconostoc*. Based on LAB identification by API 50 CHL test strips, the identified LAB species include *Lactobacillus acidophilus* (2 isolate*), Lactiplantibacillus plantarum* (1 isolate), and *Limosilactobacillus fermentum* (2 isolate). Our study finding is consistent with study conducted in India [29] with isolation and identification of similar Lactobacillus species. Lactobacilli are extensively investigated in the food industry due to their beneficial effects. According to current taxonomic classification, *Lactobacillus plantarum* and *Lactobacillus fermentum* were renamed as *Lactiplantibacillus plantarum* and *Limosilactobacillus fermentum*, respectively [41, 42]. In our work, *Lactococcus lactis* subsp. *lactis* (6 isolates) and *Leuconostoc lactis* (1 isolate) were identified. This finding is similar to a study conducted in Ethiopia [27] with isolation and identification of similar *Lactococcus lactis* subsp. *lactis* and *Leuconostoc lactis* species. In our study finding, mesophilic lactic acid bacteria were dominantly isolated and identified. The reason could be sampling season (samples were collected during the cooler months of May-July; the ambient temperature at which the natural fermentation of the tested samples occurred and allowed the proliferation of mesophilic lactic acid bacteria [43]). Fermentation of food products by LAB is also dependent on climatic conditions of the area where fermentation occurred [37].

One of most important/desirable properties of LAB, as starter culture or probiotics, is their antimicrobial activity [44, 45]. During primary screening of LAB, LAB isolate ESCIa-9 showed strong antimicrobial activity against *S. aureus* with an inhibition zone of 13.6 ± 3.1 mm, whereas ESBIa-5 showed strong antimicrobial activity against *E. coli* with an average inhibition zone of 12 ± 1.8 mm, while ESCIc-11 exhibited strong antimicrobial activity against *Salmonella* species with an average inhibition zone of 11.6 ± 3.6 mm. This finding is similar to study findings conducted in Indonesia by Prihanto et al. [46] on fermented fish (peda) isolates with a diameter of the inhibition between 10-20 mm.

Based on the result of secondary screening of LAB, the higher antagonistic activity of CFS of LAB isolate against *S. aureus* and *E. coli* was 14.12 ± 1.6 mm and 13.5 ± 2.1 mm, respectively. As observed here, the present study result showed a good inhibition zone against *S. aureus* when compared to the study conducted in Gondar town by Lelise et al. [33] and in Ngaoundere, Cameron, by Mbawala et al. [34], who reported 12.3 ± 1.6 mm and 4.5 ± 0.1 mm as the maximum inhibition zone of CFS of LAB against *S. aureus*, respectively. The finding of this study also reflects a good inhibition zone against *E. coli* when compared with studies conducted in Cameron [34] and Malaysia [47], which reported 4.5 ± 0.1 mm and 1.3 ± 0.5 mm as the maximum inhibition zone of lactic acid bacteria against *E. coli*. Our study findings showed that the maximum antagonistic activity of CFS of LAB isolates against S*almonella* species was 12.9 ± 3.6 mm, which is higher than study findings from Indonesia studied by Sari et al. [48] and in Mosul, Iraq, by Al-Allaf et al. [35], who reported 7.5 mm and 0.75 mm as the maximum inhibition zone of lactic acid bacteria against *Salmonella* species, respectively. Our result indicated slightly higher activity compared to research findings from Indonesia reported by Prihanto et al. [46], where the maximum inhibition zone of LAB against *Salmonella* species was 10.3 mm. Based on the average zones of inhibition produced by LAB isolates, *Lactobacillus* isolates showed the highest inhibition to the test bacteria, followed by *Lactococcus* and *Leuconostoc* isolates. This finding is similar to a study conducted in Ethiopia by Amenu [49], who reported that *Lactobacillus* isolates showed the highest inhibition to the test bacteria.

Among the indicator/test bacteria, *Staphylococcus aureus* was highly sensitive to LAB isolates followed by *E. coli* and *Salmonella* species. It was indicated that the highest sensitivity of *S. aureus* to the antimicrobial effect of LAB is due to the reason that antimicrobial compounds are effective for related genera, as indicated by Timothy et al. [50]. Based on secondary screening, CFS of LAB isolates was heat resistant at various temperatures. This might be due to the thermostable nature of LAB bacteriocins [51]. This finding is also supported by another study conducted in Egypt by Pato et al. [52], which indicated that bacteriocin is resistant to high temperature. This is because they are proteinaceous in nature and have low molecular weight with diversified secondary structures. However, antimicrobial activity of CFS is reduced when the temperature is increased [14]. Inhibitory activity of CFS is reduced after treatment by enzymes such as pepsin and trypsin. This is due to the proteinaceous nature of antimicrobial compounds [22]. Additionally, AMCds of LAB (e.g., bacteriocin) are easily inactivated by stomach-related protein-degrading enzymes and have little impact on the gut microbiota [19].

But, AMA of CFS was not affected by amylase enzymes. Similarly, Savadogo et al. [39] and Mezaini et al. [53] indicated that antimicrobial activity of CFS was not inactivated after treatment with amylase enzyme. This might be due to the proteinaceous nature of antibacterial molecule in CFS. On the other hand, Savadogo et al. [39] mentioned that antibacterial activity of CFS for some LAB is not only inactivated by protein-degrading enzymes but also by other carbohydrate-degrading enzymes. During primary screening, for three indicator bacteria such as *S. aureus*, *E. coli*, and *Salmonella* species, average zones of inhibition statistically differ from each other in response to antimicrobial effect by some LAB isolates such as ESAIb and ESCIa with *P* < 0.05. However, there were no other significant differences found between other conditions *P* > 0.05.

## 5. Conclusion and Recommendation

Lactic acid bacteria (LAB) were isolated and identified from three different samples of fermented milk (Ergo). The dominant isolates of LAB belong to *Lactococcus lactis* subsp. *lactis* followed by *Lactobacillus acidophilus, Limosilactobacillus fermentum, Lactiplantibacillus plantarum*, and *Leuconostoc lactis* species, respectively. The LAB isolates under investigation showed antimicrobial effects against indicator/test bacteria such as *S. aureus*, *E. coli*, and *Salmonella* species. Among test bacteria, *S. aureus* was highly sensitive to the antimicrobial effects of LAB. *Escherichia coli* and *Salmonella* species showed moderate sensitivity with smaller inhibition zone to antimicrobial effects of LAB. *Lactobacillus* isolates exhibited the highest antagonistic activity against indicator bacteria.

Based on the obtained result and conclusion of this study, the following recommendations will be proposed: since LAB isolates belonging to *Lactobacillus* genera showed the highest antimicrobial activity against indicator bacteria, additional investigations are proposed, such as analysis of probiotic features and *in vivo* probiotic effects of those LAB isolates. The presence of LAB and antimicrobial molecules in fermented milk allows/ensures that the milk is safe for consumption, representing a promising agent in the future as a biological antimicrobial agent for food biopreservation in the food industry. Since the increasing use of antibiotics leads to collateral damage to the host by disturbing the normal intestinal microbiota; therefore, consumption of fermented foods such as fermented milk enhances the proliferation of healthy GIT microbiota on the one hand and prevents the growth of undesirable microorganisms.

## Figures and Tables

**Table 1:** Morphological characteristics of LAB isolated from Ergo samples, Jimma town, southwest Ethiopia.

S. no	LAB isolate code	LAB genus detected	Gram stain	Cell morphology	Cellular arrangement/appearance
1	ESAIa	Lactococcus	+	Cocci	Single, diploid, and short-chained cocci
2	ESAIb	Lactococcus	+	Cocci	Tetraploid, paired cocci
3	ESAIc	Lactococcus	+	Cocci	Paired, long-chained cocci
4	ESAId	Lactococcus	+	Cocci	Paired, short-chained cocci
5	ESBIa	Lactobacillus	+	Rod	Paired rods
6	ESBIb	Leuconostoc	+	Cocci	Long-chained cocci
7	ESBIc	Lactococcus	+	Cocci	Diploid, paired cocci
8	ESBId	Lactococcus	+	Cocci	Single, paired, and chained cocci
9	ESCIa	Lactobacillus	+	Coccoid bacilli	Paired, long-chained coccoid bacilli
10	ESCIb	Lactobacillus	+	Coccoid bacilli	Single, paired coccoid bacilli
11	ESCIc	Lactobacillus	+	Coccoid bacilli	Diploid, tetraploid, and paired coccoid bacilli
12	ESCId	Lactobacillus	+	Coccoid bacilli	Single, diploid, and paired coccoid bacilli

**Table 2:** Biochemical and physiological characteristics of LAB isolated from Ergo samples, Jimma town, southwest Ethiopia.

S. no	LAB isolate code	Characteristics tested											
		CAT	TSIA		Ctr	Gas production	Growth at d/nt temperature			Growth at d/nt NCl conc			LAB genus detected
			Y/Y	H_2_S			10°C	15°C	45°C	2%	4%	6.5%	
1	ESAIa	—	+	—	—	—	+	+	—	+	+	—	Lactococcus
2	ESAIb	—	+	—	—	—	+	+	—	+	+	—	Lactococcus
3	ESAIc	—	+	—	—	—	+	+	—	+	+	—	Lactococcus
4	ESAId	—	+	—	—	—	+	+	—	+	+	—	Lactococcus
5	ESBIa	—	+	—	—	+	—	+	+	+	+	+	Lactobacillus
6	ESBIb	—	+	—	—	+	+	+	—	+	+	+	Leuconostoc
7	ESBIc	—	+	—	—	—	+	+	—	+	+	—	Lactococcus
8	ESBId	—	+	—	—	—	+	+	—	+	+	—	Lactococcus
9	ESCIa	—	+	—	+	+	—	+	+	+	+	+	Lactobacillus
10	ESCIb	—	+	—	+	+	—	+	+	+	+	+	Lactobacillus
11	ESCIc	—	+	—	—	+	—	+	—	+	+	+	Lactobacillus
12	ESCId	—	+	—	—	+	—	+	+	+	+	+	Lactobacillus

Legend: LAB: lactic acid bacteria, CAT: catalase, “+” means positive reaction, “-” means negative, d/nt: different, conc: concentration, Ctr: Simmon citrate, ESA: Ergo sample one, ESB: Ergo sample two, ESC: Ergo sample three, Ia: isolate one, Ib: isolate two, Ic: isolate three, and Id: isolate four.

**Table 3:** Identification of LAB isolates by using API 50 CHL test strips.

S. no	LAB isolates	Sugars											
		Arab	Gala	Inu	La	Malt	Manni	Sucr	Xyl	Sori	Ribo	Raff	Meli
1	ESAIa	+	+	—	+	+	+	Wp	—	—	+	—	+
2	ESAIb	+	+	—	+	+	+	+	—	—	+	—	+
3	ESAIc	+	+	—	+	+	+	+	—	—	+	—	+
4	ESAId	+	+	—	+	+	+	+	—	—	+	—	—
5	ESBIa	—	+	—	+	+	—	+	+	+	—	+	—
6	ESBIb	+	+	—	+	+	NCc	+	+	—	—	—	+
7	ESBIc	+	+	—	+	+	+	+	—	—	+	—	+
8	ESBId	+	+	—	+	+	+	+	—	—	+	—	+
9	ESCIa	—	+	—	+	+	—	+	+	+	—	+	—
10	ESCIb	+	+	—	+	+	+	+	+	+	+	+	+
11	ESCIc	+	+	+	+	+	+	+	+	—	+	+	+
12	ESCId	+	+	+	+	+	+	+	+	—	+	+	+

Legend: Arab: arabinose, Gala: galactose, Inu: inulin, La: lactose, Malt: maltose, Manni: mannitol, Sucr: sucrose, Xyl: xylose, Sori: soribitol, Ribo: ribose, Raff: raffinose, and Meli: melibiose. Wp: weakly positive; NCc: no color change.

**Table 4:** ANOVA SPSS output of LAB isolated from Ergo samples, Jimma town, southwest Ethiopia.

ANOVA						
		Sum of squares	Df	Mean square	*F*	Sig.

Zone of inhibition in mm by LAB isolate 1	Between groups	1.500	2	.750	.036	.965
	Within groups	188.750	9	20.972		
	Total	190.250	11			

Zone of inhibition in mm by LAB isolate 2	Between groups	181.167	2	90.583	6.548	**.018**
	Within groups	124.500	9	13.833		
	Total	305.667	11			

Zone of inhibition in mm by LAB isolate 3	Between groups	13.167	2	6.583	.560	.590
	Within groups	105.750	9	11.750		
	Total	118.917	11			

Zone of inhibition in mm by LAB isolate 4	Between groups	3.167	2	1.583	.152	.861
	Within groups	93.500	9	10.389		
	Total	96.667	11			

Zone of inhibition in mm by LAB isolate 5	Between groups	50.667	2	25.333	1.407	.294
	Within groups	162.000	9	18.000		
	Total	212.667	11			

Zone of inhibition in mm by LAB isolate 6	Between groups	96.167	2	48.083	3.504	.075
	Within groups	123.500	9	13.722		
	Total	219.667	11			

Zone of inhibition in mm by LAB isolate 7	Between groups	50.167	2	25.083	1.727	.232
	Within groups	130.750	9	14.528		
	Total	180.917	11			

Zone of inhibition in mm by LAB isolate 8	Between groups	69.500	2	34.750	4.088	.055
	Within groups	76.500	9	8.500		
	Total	146.000	11			

Zone of inhibition in mm by LAB isolate 9	Between groups	165.125	2	82.563	8.697	**.008**
	Within groups	85.438	9	9.493		
	Total	250.563	11			

Zone of inhibition in mm by LAB isolate 10	Between groups	96.167	2	48.083	3.644	.069
	Within groups	118.750	9	13.194		
	Total	214.917	11			

Zone of inhibition in mm by LAB isolate 11	Between groups	27.125	2	13.563	1.279	.324
	Within groups	95.438	9	10.604		
	Total	122.563	11			

Zone of inhibition in mm by LAB isolate 12	Between groups	43.167	2	21.583	1.139	.362
	Within groups	170.500	9	18.944		
	Total	213.667	11			

**Table 5:** Multiple comparison of average inhibition zone of LAB isolated from Ergo samples, Jimma town, southwest Ethiopia.

Multiple comparisons				
Tukey HSD				

Dependent variable	(I) Test strains	(J) Test strains	Mean difference (I-J)	Sig.

Zone of inhibition in mm by LAB isolate 1	*S. aureus*	*E. coli*	.0000	1.000
		*Salmonella* spp.	.7500	.971
	*E. coli*	*S. aureus*	.0000	1.000
		*Salmonella* spp.	.7500	.971
	*Salmonella* spp.	*S. aureus*	-.7500	.971
		*E. coli*	-.7500	.971

Zone of inhibition in mm by LAB isolate 2	*S. aureus*	*E. coli*	9.5000^∗^	**.014**
		*Salmonella* spp.	5.2500	.169
	*E. coli*	*S. aureus*	-9.5000^∗^	**.014**
		*Salmonella* spp.	-4.2500	.288
	*Salmonella* spp.	*S. aureus*	-5.2500	.169
		*E. coli*	4.2500	.288

Zone of inhibition in mm by LAB isolate 3	*S. aureus*	*E. coli*	2.5000	.577
		*Salmonella* spp.	.7500	.949
	*E. coli*	*S. aureus*	-2.5000	.577
		*Salmonella* spp.	-1.7500	.757
	*Salmonella* spp.	*S. aureus*	-.7500	.949
		*E. coli*	1.7500	.757

Zone of inhibition in mm by LAB isolate 4	*S. aureus*	*E. coli*	-.5000	.974
		*Salmonella* spp.	-1.2500	.850
	*E. coli*	*S. aureus*	.5000	.974
		*Salmonella* spp.	-.7500	.942
	*Salmonella* spp.	*S. aureus*	1.2500	.850
		*E. coli*	.7500	.942

Zone of inhibition in mm by LAB isolate 5	*S. aureus*	*E. coli*	-3.0000	.595
		*Salmonella* spp.	2.0000	.788
	*E. coli*	*S. aureus*	3.0000	.595
		*Salmonella* spp.	5.0000	.269
	*Salmonella* spp.	*S. aureus*	-2.0000	.788
		*E. coli*	-5.0000	.269

Zone of inhibition in mm by LAB isolate 6	*S. aureus*	*E. coli*	2.0000	.733
		*Salmonella* spp.	6.7500	.070
	*E. coli*	*S. aureus*	-2.0000	.733
		*Salmonella* spp.	4.7500	.220
	*Salmonella* spp.	*S. aureus*	-6.7500	.070
		*E. coli*	-4.7500	.220

Zone of inhibition in mm by LAB isolate 7	*S. aureus*	*E. coli*	1.0000	.928
		*Salmonella* spp.	4.7500	.236
	*E. coli*	*S. aureus*	-1.0000	.928
		*Salmonella* spp.	3.7500	.385
	*Salmonella* spp.	*S. aureus*	-4.7500	.236
		*E. coli*	-3.7500	.385

Zone of inhibition in mm by LAB isolate 8	*S. aureus*	*E. coli*	1.7500	.684
		*Salmonella* spp.	5.7500	**.050**
	*E. coli*	*S. aureus*	-1.7500	.684
		*Salmonella* spp.	4.0000	.183
	*Salmonella* spp.	*S. aureus*	-5.7500	.050
		*E. coli*	-4.0000	.183

Zone of inhibition in mm by LAB isolate 9	*S. aureus*	*E. coli*	6.1250^∗^	**.049**
		*Salmonella* spp.	8.8750^∗^	**.007**
	*E. coli*	*S. aureus*	-6.1250^∗^	**.049**
		*Salmonella* spp.	2.7500	.449
	*Salmonella* spp.	*S. aureus*	-8.8750^∗^	.**007**
		*E. coli*	-2.7500	.449

Zone of inhibition in mm by LAB isolate 10	*S. aureus*	*E. coli*	6.7500	.065
		*Salmonella* spp.	2.0000	.725
	*E. coli*	*S. aureus*	-6.7500	.065
		*Salmonella* spp.	-4.7500	.209
	*Salmonella* spp.	*S. aureus*	-2.0000	.725
		*E. coli*	4.7500	.209

Zone of inhibition in mm by LAB isolate 11	*S. aureus*	*E. coli*	3.2500	.376
		*Salmonella* spp.	.1250	.998
	*E. coli*	*S. aureus*	-3.2500	.376
		*Salmonella* spp.	-3.1250	.402
	*Salmonella* spp.	*S. aureus*	-.1250	.998
		*E. coli*	3.1250	.402

Zone of inhibition in mm by LAB isolate 12	*S. aureus*	*E. coli*	4.2500	.390
		*Salmonella* spp.	.5000	.986
	*E. coli*	*S. aureus*	-4.2500	.390
		*Salmonella* spp.	-3.7500	.472
	*Salmonella* spp.	*S. aureus*	-.5000	.986
		*E. coli*	3.7500	.472

^∗^The mean difference is significant at the 0.05 level.

**Table 6:** Antimicrobial activity of heat-treated CFS.

LAB isolates	Average ZOI of CFS after treatment with heat			
	30°C	60°C	80°C	100 °C
ESBIa	9.3 ± 3.2	7.7 ± 2.2	6.7 ± 2.1	3 ± 0.4
ESCIa	10.2 ± 2.2	8.7 ± 4.2	7.2 ± 3.5	6.5 ± 1.2
ESCIc	11.7 ± 4.7	9.5 ± 5.7	7.7 ± 4.1	4 ± 3.1

Results expressed as average of (*n* = 3) ± SD (standard deviation).

**Table 7:** Antimicrobial activity of enzyme-treated CFS.

LAB isolates	Average ZOI of CFS after treatment with enzyme		
	Amylase	Trypsin	Pepsin
ESBIa	9.3 ± 3.2	6 ± 4.1	^∗^
ESCIa	10.2 ± 2.2	^∗^	7.5 ± 1.5
ESCIc	11.7 ± 4.7	10 ± 1.2	^∗^

Results expressed as average of (*n* = 3) ± SD (standard deviation). Legend: ^∗^zone of inhibition not observed.

**Table 8:** Antimicrobial susceptibility pattern of indicator bacteria.

Clinical isolate (N = 12)		Antimicrobial agents (*n* (%))					
		AMP	CZO	TCY	GEN	CRO	CIP
*E. coli*, *n* = 4	S	2 (50)	1 (25)	3 (75)	0 (0)	1 (25)	1 (25)
	I	0 (0)	0 (0)	0 (0)	0 (0)	0 (0)	0 (0)
	R	2 (50)	3 (75)	1 (25)	4 (100)	3 (75)	3 (75)

		AMP	CRO	CAZ	IMI	MEM	TCY
*Salmonella* spp., *n* = 4	S	0 (0)	4 (100)	0 (0)	4 (100)	0 (0)	4 (100)
	I	0 (0)	0 (0)	0 (0)	0 (0)	0 (0)	0 (0)
	R	2 (50)	3 (75)	1 (25)	4 (100)	3 (75)	3 (75)

		CLN	ERY	PN	TCY	DOX	GEN
*S. aureus*, *n* = 4	S	4 (100)	2 (50)	1 (25)	2 (50)	3 (75)	4 (100)
	I	0 (0)	1 (25)	0 (0)	1 (25)	1 (25)	0 (0)
	R	0 (0)	1 (25)	3 (75)	1 (25)	0 (0)	0 (0)

Source of AST pattern (unpublished raw data which was taken from Jimma University Medical Center, Medical Microbiology Laboratory).

## Data Availability

All data generated or analyzed during this study are included in this manuscript, except few sensitive information like names of the milk sellers linked to their results.

## Abbreviations

LAB: Lactic acid bacteria
FAO: Food and agricultural organization
WHO: World Health Organization
ISAPP: International scientific association for probiotics and prebiotics
MRS: de Mann Rogosa Sharpe agar
ZOI: Zone of inhibition
ATCC: American type culture collection
CFS: Cell-free supernatant
AMA: Antimicrobial activity
AMCpds: Antimicrobial compounds
API: Analytical profile index.
